# Critical steps to tumor metastasis: alterations of tumor microenvironment and extracellular matrix in the formation of pre-metastatic and metastatic niche

**DOI:** 10.1186/s13578-020-00453-9

**Published:** 2020-07-28

**Authors:** Jianan Zhuyan, Mingyu Chen, Tianhao Zhu, Xunxia Bao, Timing Zhen, Kaichen Xing, Qiubo Wang, Sibo Zhu

**Affiliations:** 1 School of Life Sciences, Fudan University, 2005 Songhu Road, Shanghai, 200438 China; 2 Shanghai Starriver Bilingual School, Shanghai, 201100 China; 3 Department of Neurosurgery, Huashan Hospital, Shanghai, 200040 China; 4 School of Medical Instrument and Food Engineering, University of Shanghai for Science and Technology, Shanghai, 200093 China; 5 Department of Clinical Laboratory, Wuxi 9th Affiliated Hospital of Soochow University, No.999 Liangxi Road, Wuxi, China

**Keywords:** ECM components, Metastasis, Organ specificity, Mechanism, Systematic literature review

## Abstract

For decades, cancer metastasis has been a heated topic for its high mortality. Previous research has shown that pre-metastatic niche and metastatic niche are the 2 crucial steps in cancer metastasis, assisting cancerous cells’ infiltration, survival, and colonization at target sites. More recent studies have unraveled details about the specific mechanisms related to the modification of pro-invasion environments. Here, we will review literatures on extracellular matrix (ECM) alterations, general cancer metastasis, organ specificity, pre-metastatic niche, metastatic niche, colony formation and impact on the course of metastasis. Respectively, the metastatic mechanisms like effect of hypoxia or inflammation on pre-metastatic niche construction, as well as the interaction between cancer cells and local milieu will be discussed. Based on the evidences of metastatic niches, we revisit and discussed the “Seed and Soil” hypothesis by Paget. This review will seek to provide insight into the mechanism of metastatic organ specificity which pre-metastatic niche and metastatic niche might suggest from an evolutionary aspect.

## Introduction

Tumors often manifested metastasis during its development: an aggressive and tricky move which caused over 90% of cancer-related deaths [1]. Whereas amounts of cancer types exhibit metastatic phenotype, the metastatic sites often vary with respect to the primary site (Table 1). Even though cancer cells metastasize to the same foci, the microenvironment at the target organ still poses a distinctive challenge for a variety of primary cancer types. Thus, finding out how tumor cells survive and colonize in the adverse microenvironment is crucial to our understanding of metastasis.

**Table 1:** Cumulative metastasis rate in various cancer types

Primary organs	Incidence rate	Target organ
Breast cancer	21–32% [78,79]	Lung
Melanoma	18–36% [80,81]	
Colorectal cancer	10–15% [82,83]	
Sarcoma	20–25% [84,85]	
Breast cancer	4–30% [78,79]	Brain
Lung cancer	23–36% [86]	
Melanoma	5.8–28% [87–89]	
Renal cancer	6.5–11% [88]	
Breast cancer	50% [90]	Liver
Lung cancer	10–14% [91]	
Colorectal	35–55% [92–94]	
Pancreatic	40%–90% [95,96]	
Melenoma	14–20% [81]	
Breast cancer	30–60% [78,79]	Bone
Lung cancer	9–39% [97,98]	
Prostate cancer	68–80% [99,100]	
Melanoma	11–17% [80,81]	

Basically the process of metastasis is defined as a cascade: local invasion, intravasation, survival in the circulation, extravasation and colonization [2]. The process of colonizing the target organ, more precisely, is to colonize the target niche in the organ. Though, recent years more and more researches have shown evidence that supports the existence of “pre-metastatic niche (PMN),” the modification of microenvironment of metastatic site devoid of arrival of circulating tumor cells (CTCs), since the first identification of it in 2005 by Kaplan [3,4].

Modifying niches and interacting with local milieu, being able to form pre-metastatic niche and colonize the metastatic niche, therefore become important abilities for cancer cells to possess in order to achieve higher fitness in an environment hostile to them.

## Preconditioning the niche: an ECM matter

Various specific pathological conditions can induce microenvironment change, leading to different outcomes include regulation of expression of proteins and structural changes. As a comprehensive co-effort potential mechanisms, the extracellular matrix (ECM) is changed due to various reasons in the formation of PMN. ECM change is one of the most significant pre-metastatic changes on the target foci. The present-day investigation on PMN showed that the formation of PMN supports the cancer cell engraftment. It can be modified through the recruitment of various types of cells, altered expression of matrix proteins, and properties of ECM. Fibroblasts play a key role in depositing ECM protein and remodeling. Growth factors and chemokines produced by endothelial cells when cancer occurs can promote T lymphocyte infiltration, macrophage activation, and fibroblast differentiation into cancer-associated fibroblasts (CAF) [5]. CAF through matrix metalloproteinase (MMP), RhoA, ROCK, non-muscle myosin-II (MyoII), and palladin modify the ECM generating a niche that supports cancer cell invasion [6]. During the progression of cancer, the imbalance of ECM's homeostasis will profoundly affect the function of tumor cells. ECM components mainly include fibronectin, versican, collagen I/III/IV, TGF-β, and periostin. In various cases, bone marrow-derived cells (BMDCs) are recruited to target site in response to accumulated fibronectin. For pancreatic cancer that metastasize to liver, Kupffer cells in liver could be stimulated by pancreas derived cell through MIF, further induce hepatic stellate cells (HSCs) to deposit fibronectin at ECM, eventually summon BMDCs and trigger the PMN formation [7]. Hepatocytes can also increase the production of serum amyloid A1 and A2 (SAA) by activating IL-6/STAT3 signaling, thereby changing the liver's immune and fibrotic microenvironment, thus establishing PMN [8]. Both BMDC-derived EVs and miR-92a mimics potentiate the activation of HSCs, subsequently increasing ECM deposition and regulates hepatic PMN in lung cancer [9]. It has been found in various breast cancer models that versican is involved in tumor occurrence and metastasis. Experiment have shown that elevated levels of PAPSS2 and versican are essential for snail-mediated breast cancer cell migration and metastasis [10]. Tumor associate macrophages (TAMs) participate in the regulation of murine signaling 4T1 breast cancer mode by versican implies the potential of versican as an attractive target for breast cancer therapy [11]. It has also been found that TAM directly promotes tumor niche formation and participates in the deposition of ECM collagen fibers by producing MMP-2, MMP-9 and matrix-related proteins [12]. Another structural protein, periostin, induced change is shown in mouse models of breast cancer, when several factors like TGF-β up-regulated the expression of αSMA and VIM in lung, supporting the successful infiltration of malignant cells through WNT signal pathway [12,13]. The LOX results in increased tissue stiffness of ECM. This change in turn supports cancer cells’ extravasation through compromising the tissue function as well as induce enhancement of PI3 Kinase (PI3K) activity through focal adhesion by promoting the Akt signaling pathway [14,15]. LOXL2 enhances adhesion signals by stabilizing the expression of integrin α5β1, and activates CAF through FAK activation mediated by β3 integrin, which have long been shown crucial to promote tumor invasion and progression [16]. BMDC is also recruited under LOX regulating, having related to the collagen cross-linking process. Those BMDCs shares a positive feedback loop with expression of MMP, as both stimulate the expression or recruitment of the other, eventually leads to the pro-invasion and pro-colonization microenvironment [17,18]. Other functions of BMDC included that it could secrete versican which stimulated mesenchymal to epithelial transition (MET), aiding the metastatic process [19].

## Pathological syndromes induced in PMN formation

**Fig. 1:**
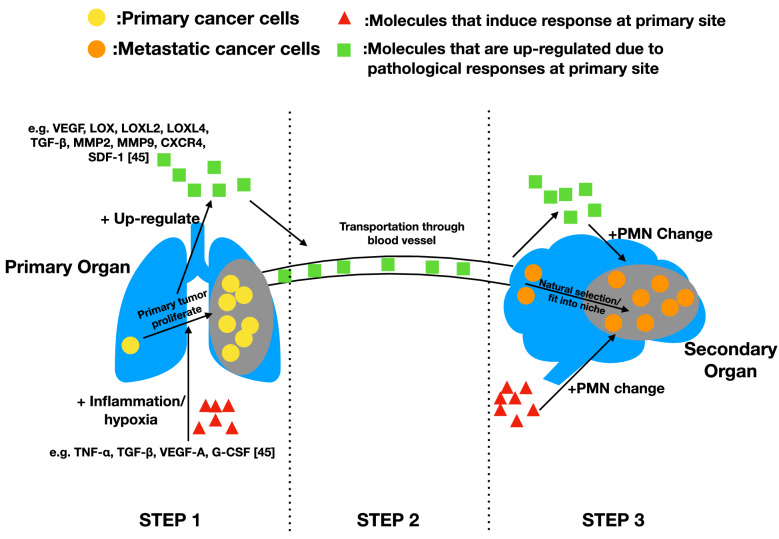
Primary tumor induced PMN change. This figure shows the process of how primary organ responses directed PMN formation. STEP 1: Some molecules at primary site induce response like inflammation and hypoxia at primary site. These responses assist the proliferation of primary tumor and up-regulates expression of certain molecules. STEP 2: Those molecules are transported from primary site to secondary site through blood vessel. STEP 3: Those molecules induce PMN formation at secondary site through various approaches. Also, some of the molecules that induce response at primary site functioned similarly at secondary site, inducing similar responses that affect PMN formation. The change of PMN built a unique environment which favored metastatic tumor cells for colonization. This figure shows that many molecules and pathways are similar at both sites during primary tumor proliferation and PMN formation, suggesting that PMN formation could be a byproduct of primary tumor growth, and the organ specificity of metastasis might lie within the similarity between organs

The alteration of physical structure of tissue is extremely important in malignant diseases, such as cancer metastasis, as it directly affects the extravasation and colonization of CTCs [20]. Even though investigations about the exact mechanisms of how ECM structurally affect metastasis are still in infancy, general syndromes that are induced in PMN formation can be characterized as more comprehensive and integrated aspects on this issue. Here, two pathological syndromes, inflammation and hypoxia, are concluded (Fig. 1).

### Suppressed immunity and triggered inflammation

Triggering abnormal immune responses through inflammation at target site is a common and effective strategy, preparing for a full-grown metastasis. In this process, various chemical signal is altered in a network aimed to normalize, or “heal” the tissue while local environment is disturbed, as different cells are recruited to ECM, forming a pre-metastatic niche [21].

The interaction between tumor cells and immune cells is extremely important in the process of tumor metastasis. Monocytes, neutrophils, and macrophages are the main components of the microenvironment of metastatic tumors, affecting the recruitment and extravasation of tumor cells before metastasis. ANGPTL2 initiates programs that lead to neutrophil recruitment within the lung, a process essential for the lung colonization in spontaneous models of osteosarcoma pulmonary metastasis [22]. Hyunho Kim et al. developed a microfluidic platform that incorporates endothelial cells and extracellular matrix scaffolds, proved monocyte‐derived matrix metalloproteinase 9 facilitates cancer cell extravasation through destruction of endothelial tight junctions, and macrophages could reduced migratory capacity of cancer cells [23]. Immune cells such as NK cells, CD8 + T cells, and interleukin-1β-expressing innate immune cells can impair metastatic niche development. Accumulation of Tregs around colon carcinoma MC38 liver micrometastases promotes an immunosuppressive microenvironment in the liver to promote the colonization and growth of hepatic metastases. The inhibitory effects of pancreatic cancer derived extracellular vesicles on NK cells represent a mechanism allowing metastatic tumor cells to escape from NK cell immune surveillance in the pre-metastatic niche [24]. Zafira Castaño et al. discovered that IL-1β preventing breast cancer cells from generating highly proliferative E-cadherin-positive progeny at the metastatic site, thereby overt metastases cannot be established [25].

Lots of studies have shown that inflammatory chemokine induced regional inflammation at the metastatic site, such as up-regulating expression of S100 proteins like S100A8 and S100A9 through release of VEGF-A, TGF-β and TNF-α in lung cancer exclusively [4,21,26–29]. This process resulted in the recruit of Mac-1 (CD11b/CD18) + myeloid cells that is hypothesized to make the milieu at metastatic site to be immunosuppressive, and the collagenase actively enabled DTCs to breakthrough.[26,30]. Genes like CCL2 and receptor like TLR4 also cause inflammation prior to the metastasis, recruiting leukocyte activity and increasing vascular permeability, allowing both immune-compromise and an easier path for extravasation of CTC [31–33]. In metastasis to liver, excessive TIMP-1 leads to binding of SDF-1, triggering inflammation and further immunocompromise as a part of modification.[34,35]. Moreover, in infiltration to brains, blood–brain barrier’s (BBB) physical structure could be disturbed through a triggered inflammatory response, making CTCs easier to break through.

### Hypoxia

Another approach of establishing is hypoxia, which is common in cancerous disease due to angiogenesis and growth of tumor cells, which results in a heterogeneous distribution of oxygen and nutrient in tumorous sites [36]. Hypoxia can be induced in both primary site and metastatic site during PMN formation, and it’s the mutual contribution of hypoxia in both site leads to further modification of PMN.

Hypoxia is a special environment in which genes are regulated differently, and it contributed to imitate and prepare for a successful metastasis. Hypoxia can be induced at secondary metastatic site as a part of pre-metastatic niche. In liver metastasis, it is reported that the previously mentioned TIMP-1 leads to up-regulation of Hypoxia-inducible factor -1 (Hif-1), which in turn induce hypoxia at target site, making it more vulnerable to metastasis [37]. In other studies, both LOX secreted in primary site and the one delivered to secondary site is shown to be responsible for the osteolytic lesion in bones as well as recruitment of BMDC by cross-linking collagen in lungs and other organs, supporting colonization and proliferation of cancerous cells [17,38–40]. Hypoxia at primary organ also induces the release of exosome, which is crucial to the generation of PMN [41].

## Exosomes

Exosomes play a critical role in the development of pre-metastatic niches and the mechanism is extremely complex. Exosomes can promote the metastasis of many types of tumors through the fuction of communication medium. Studies on head and neck cancer by *Ludwig S *et al*.* have shown that exosomes can impaired function of T cells, NK cells, and antigen-presenting cells, and thereby forming an immunosuppressive premetastatic microenvironment [42]. Ovarian cancer exosomes can promote the proliferation and migration of tumor cells in the pre-metastatic niche by inducing TAM [43]. The role of exosomes is controversial in some melanoma studies. *Shin La Shu *et al*.* found that metabolic reprogramming of stromal fibroblasts by melanoma exosome microRNA favours a pre-metastatic microenvironment [44]. However, the research by *Michael P. Plebanek *et al*.* puts forward the opposite view, they demonstrates that pre-metastatic melanoma tumors produce exosomes, which elicit a broad range of PMo-reliant innate immune responses via trigger of immune surveillance and potently inhibit metastasis to the lung [45]. In addition, exosomes also play an irreplaceable role in increasing angiogenesis and vascular permeability in the pre-metastatic niche. *Hoshino *et al*.* explored the organ specificity of exsomes in terms of integrins that α6β4 and α6β1 were associated with lung metastasis, while αvβ5 was linked to liver metastasis. The integrins were further shown to have the ability to activate Src phosphorylation and pro-inflammation S100 family to form the metastasis niche [45].

## Interaction of CTC and metastatic niche

Metastatic niche is where the CTCs land after circulating and begin the colonization process. However, this process is harsh for cancer cells, as the metastatic site is already an established microenvironment. Thus, for CTCs, the only approach in fit in the local milieu is to fit in the a niche, either compete with existing natives or create a new niche, through cross-talks and interaction with the local cells. Intriguing, utilizing local components and molecules in the course of cell–cell interaction is a common way for CTCs to gain most benefit, and it could be seen as a “rule of expediency” as it provides a ready-made tool for invading cells to survive and proliferate.

CTCs as potential metastatic seeds can form metastatic sites in either singleton or cluster way [46]. CTC clusters are shown to have 50-fold increased metastatic potential compared to the single cell form [47]. This is achieved through the higher expression of cell junction component, i.e. plakoglobin. CTC cluster can also form tumor microemboli, being associated with poorer prognosis [48]. Roles of CTCs are deciphered as the development of single-cell RNA-seq technique [49]. This approah combined with staining-based microscopy or flow cytometry provides us a more comprehensive understanding of the mechanisms of CTCs including metastasis, and cancer stemness. Formation of metastatic sites relies on adhesion of CTCs to the endothelial cells. This process depends not only on the adhesion receptors of CTCs but also on the receptor repertoire of accompanying cells or fractions such as neutrophils and platelets [50]. As soon as the CTCs adhere to the endothelia in the target organs, recruitment of platelet and granulocytes promotes the early metastatic niches. This process relies on platelet-derived CXCL5/7 chemokines [51].

## Metastasis niches in multiple organs

CTCs overcome obstacles and spread to distant organs to survive through multiple mechanisms. The ECM of distant organs can be remodeled in a way that promotes the implantation of metastatic cancer cells, allowing them to colonize at these sites and establish metastasis. This complex process often involves loss of cell-to-cell and cell-to-matrix adhesion, epithelial-to-mesenchymal transition (EMT), acquisition of a motile and invasive phenotype, intravasation, and ability to survive in circulation. Here, a brief overview of niches and interactions at different sites is provided.

### Brain niche

Brain niche is the most intriguing one among divergent metastatic niche. On one hand, brain is the most important site for human and therefore poses a most challenging situation for metastatic cancers. However, on the other hand, the well-established brain structure and properties might be utilized by malignant cells once they fit into the niche [52]. Compare to other sites of metastasis in human body, brain metastatic site is the most distinctive one, with unique ECM components, local parenchymal cells and signaling molecules [52]. Perivascular niche is the common metastasis niche in brain, and a direct contact to the brain micro-vessel is crucial, even mandatory for disseminated cancer cells to survive [52–54]. The perivascular membrane not only nourish the cancer cells with nutrient, oxygen, and survival factors, but also provides extracellular matrix proteins for possible accommodation and interaction [53].

Neurogenesis provides an important way to create a niche for growth. Perivascular nitric oxide (NO) exists in brain metastatic niche might potentiate stem cell proliferation with regard to p21Ras and MAPK pathway under hypoxic condition rather than normal condition [55,56]. Nitric oxide synthase inhibition also inhibits the brain metastasis, further proving the positive effect of neurogenesis and stemness of DTCs have on brain metastasis and niche formation [37]. The interaction with other local cells, astrocytes in brain, is also worth inspecting. Astrocytes can secrete molecules that support brain invasion, for instance, heparanase and factors that stimulate MAPK and consequently over-expresses MMP2 [57,58]. Crosstalk between astrocytes and cancer cells involves IL-6 and IL-8, which cancer cells secrete to up-regulate endothelin expression, promoting cancer progression [58]. Astrocytes are normally the glial cells that helps maintaining the homeostasis, though now they are assisting or even protecting the cancer cells, suggesting that those malignant cells might be able to utilizes local environment and compete with local cells to maximize its possibility of survival.

### Bone niche

In bone metastatic niches, a cell–cell adhesion is crucial to survival of DTCs as an instance of interaction among cells [59]. Integrins critically assist the formation of this adhesion, as αvβ3 and α4β1 are shown to promote the adhesion to ECM components [60]. Also, annexin II and its receptor involves heavily in the adhesion and communication of cancer cells with osteoblasts [61]. Those adhesions are the very first yet most critical part of a successful colonization.

Notably, some of those molecules and pathways originally promoted homeostasis and organ efficiency also potentiates the metastatic growth once the arrival of CTCs. Actually a hypothesis that suggested that the metastasis of the cancer cell to bone is similar to homing of HSCs to bone marrow in specific mechanism [62]. The molecules that participated in this mechanism included CXCL12, IL-6, annexing II and VEGF, as they contributed to both HSC and cancer cells’ infiltration and survival [3,61,63,64]. And in this way cancer cells occupy the original HSC niche, further colonize the site. The replacement of HSCs with cancer cells in the niche signifies a critical milestone in the metastatic tumor progression, as the tumor cells has gains the benefits that bodily nourishment and protection that usually grants to normal cells. This replacement is not a coincidence, but instead, an evolutionary process that involves a competition between cells, while metastatic cancer cells often wins.

### Lung niche

Cancer cells can develop a niche before metastasis to the lung by inflammatory events caused by the primary tumor before tumor cells arrive, achieved by mutual signaling between metastatic tumor cells and local non-tumor cells [33]. For example, VEGF derived from primary breast cancer cells alters the lung microenvironment before metastasis by triggering an inflammatory response and the production of prostaglandin E2, which determines that cancer cells preferentially homing to the lung [65]. The platelet ADP receptor P2Y12 recruits VEGFR1 + BMDCs and increases the deposition of ECM fibronectin in lung pre-metastatic niche, thereby selectively promoting lung metastasis [66]. The lung epithelial TLR3 can be activated by tumor exosomal RNAs to induce chemokine secretion in the lung, consequently recruiting neutrophils to the lung for pre-metastatic niche formation and promoting lung metastasis [67]. In addition, VCAM-1-expressing tumor cells acquire survival signals from macrophages in the lung pre-metastatic niche, thus promoting metastasis to the lung [68].

### Liver niche

Liver metastasis remains a major obstacle to the successful treatment of malignant diseases, especially for gastrointestinal cancers, breast cancers and melanoma. The ability of metastatic cells to survive and proliferate in the liver depends on the interactions between tumor cells and different liver-residential subsets, including sinusoidal endothelial, stellate, Kupffer and inflammatory cells [69].

Selective uptake of exosomes by Kupffer cells (KCs) in the liver causes activation of fibrotic pathways, and the establishment of a pro-inflammatory milieu that ultimately supports metastasis [7]. PDAC-derived exosomes taken up by hepatic KCs, upregulate TGFβ production, leading to increased fibronectin production by HSCs and recruitment of bone marrow-derived macrophages are essential for premetastatic niche formation [70,71]. Cancer cell interaction with Liver sinusoidal endothelial cells (LSECs) can reciprocally alter the phenotypes of both cell types, and this may lead to intravascular tumor cell destruction but can also promote metastasis through enhanced tumor cell migration and increased angiogenesis [70]. Tumor cell adhesion to hepatocytes was identified as one of the earliest events in liver metastatic potential [72]. Activated hepatic stellate cells are responsible for the production of ECM, IL-1α, VEGF, TGF-β, and angiogenic factors [65]. In addition, differences in metabolic programming determine the metastatic organ sites of tumor cells. For example, thrombopoietin promotes colorectal tumor-initiating cells (TICs) to metastasize to the pre-metastatic liver by increasing lysine catabolism in these TICs to generate glutamate for liver colonization [73].

## From PMN to MN: an evolutionary perspective on organ-specificity

Basically the niche undergoes an evolutionary process, as the microenvironment at secondary sites is altered from a place hostile to cancer cells to a place where they could engraft and proliferate [74]. The process of evolution from PMN to MN largely determined the survival and proliferation of a cancer cell. On a larger scope, usually we classify of evolutionary model of metastasis into two separate divisions: linear model and parallel model. The difference in between is that linear model describe tumor metastasis to begin after a full-grown development of primary tumor, while parallel model gives perspective that metastasis is initiated early and developed together with the primary site [75].

However, as we noticed that cells are never able to regulate themselves as they “wish” so: they are responsive unites that interact with surrounding environments and is selected by the principle of evolutionary selection. Whereas those from primary organ can’t be illuminated by a distant organ to initiate pre-metastatic niche formation on purpose, this modification of a distant site could be interpreted as a byproduct of tumor’s development the primary site [76].

As previously mentioned, conditions like inflammation or hypoxia, are integrated conditions, that involves systematic regulation of gene expression, pathways, metabolism, etc. And those conditions are prevalent among divergent pathological sites, with primary site and PMN included. Therefore those same condition triggered at both primary and secondary site also indicates that very similar pathways/genes controlled metastasis at two sites, supporting the parallel model and PMN as a byproduct.

And at the metastatic site, there are also molecules that mediates multiple functions. It could maintain the homeostasis of the site, while it could also assist malignant cells invasion and colonization. When we talked about evolutionary process in metastasis, we inevitably would mention the “Seed and Soil” model raised by Paget in 1889, which is a prophecy-like hypothesis in modern day study of nature of cancer metastasis. In this hypothesis, he proposed that both the invasive and disseminating properties of the seeds (metastatic cells) as well as the receptive and compatible microenvironment of soil (target organs) contributed in the organ-specific nature of metastasis [77].

The discovery of PMN provided a new perspective on this theory. As a byproduct of primary site development, PMN formation also exhibits organ-specific phenomena, which hints that there might be some intrinsic relationship between metastatic organ and original organ that initiates the directional modification: the byproduct shedded from primary site must be compatible to the secondary site. The MN interaction as cancer cells utilizes local resources and compete for a niche also suggested this compatibility: the ability for malignant cells from primary to fit in to a niche at secondary site.

## Conclusion

Thus, the trace of evolution from normal organ to PMN to MN might be partially predetermined or affected by the primary and secondary organs themselves, as an invisible yet intrinsic reciprocal compatibility. And the possibly involved molecules that triggers systematic responses at either site could be seen as the keys to multiple doors, while whether the molecules and mechanisms at different sites are same is yet to be certificate.

A better understanding of the mechanisms of pre-metastatic and metastatic niche formation and their characteristics will provide novel treatment strategies for the prevention and treatment of metastatic cancer. For example, checkpoint blocking therapy to activate T cell infiltration or increase NK cells to destroy metastatic niche is a promising cancer treatment. The development of a unique pre-metastatic niche biomarker to determine the extent of its formation is a reference to guide patient medication.

## Data Availability

Not applicable.

## Abbreviations

PMN: Pre-metastatic niche
CTCs: Circulating tumor cells
ECM: Extracellular matrix
CAF: Cancer-associated fibroblasts
BBB: Blood–brain barrier’s
Hif-1: Hypoxia-inducible factor -1
PI3K: PI3 Kinase
MET: Mesenchymal to epithelial transition
NO: Nitric oxide
HSCs: Hematopoietic stem cells
SAA: Serum amyloid A1 and A2
MMP: Matrix metalloproteinase
MyoII: Non-muscle myosin-II
BMDCs: Bone marrow-derived cells
TAMs: Tumor associate macrophages
KCs: Kupffer cells
LSECs: Liver sinusoidal endothelial cells
TICs: Colorectal tumor-initiating cells
